# Variation in Community Leaf Stoichiometry and Nutrient Resorption Along an Elevational Gradient on the Northern Slope of the Kunlun Mountains

**DOI:** 10.1002/ece3.72083

**Published:** 2025-08-29

**Authors:** Shuwen Xue, Atawula Jiashalaiti, Dongdong Zhang, Zhihao Zhang, Lei Li, Bo Zhang, Fanjiang Zeng, Yan Lu

**Affiliations:** ^1^ State Key Laboratory of Ecological Safety and Sustainable Development in Arid Lands Xinjiang Institute of Ecology and Geography, Chinese Academy of Sciences Urumqi Xinjiang People's Republic of China; ^2^ Xinjiang Key Laboratory of Desert Plant Roots Ecology and Vegetation Restoration Xinjiang Institute of Ecology and Geography, Chinese Academy of Sciences Urumqi People's Republic of China; ^3^ Cele National Station of Observation and Research for Desert‐Grassland Ecosystems Cele Xinjiang People's Republic of China; ^4^ University of Chinese Academy of Sciences Beijing People's Republic of China; ^5^ College of Ecology and Environment Xinjiang University Urumqi Xinjiang People's Republic of China

**Keywords:** arid areas, C:N:P stoichiometry, mountain ecosystems, nutrient resorption, plant nutrient

## Abstract

Leaf stoichiometry and nutrient resorption are key indicators for assessing nutrient‐use status and predicting nutrient limitation in plant growth. However, the patterns of variation in plant community nutrient‐use traits along elevational gradients remain unclear. To address this, we measured leaf nutrient contents of plant communities across six elevational gradients (1960 to 3548 m) on the northern slope of the Kunlun Mountains. We systematically analyzed variations in leaf stoichiometric traits, nutrient homeostasis, and nutrient resorption efficiency (NuRE), with a particular focus on the control strategies of nutrient resorption and their responses to environmental variables. The results showed that plant communities across all elevations in the study area exhibited co‐limitation by nitrogen (N) and phosphorus (P). NuRE and PRE were higher than the global average, indicating that in nutrient‐poor environments, plants adopt adaptive strategies by enhancing nutrient resorption to reduce nutrient loss. Both NuRE and PRE declined significantly at high elevations (3248–3548 m), while the NuRE:PRE ratio tended to stabilize, suggesting a reduced dependence on nutrient resorption and a more balanced N and P availability in soils at higher altitudes. NuRE was mainly regulated by plant community diversity, whereas PRE was primarily driven by climatic factors. Under the nutrient‐poor conditions of the study area, plant communities tended to adopt stoichiometric control strategies to optimize nutrient resorption, thereby enhancing the efficiency of energy and resource allocation. Furthermore, we propose that the combined use of NuRE:PRE and LTN:LTP serves as a more robust framework for assessing nutrient limitation. This study improves our understanding of the patterns of nutrient limitation and nutrient resorption processes along elevational gradients in arid mountain regions and provides new insights into nutrient regulation mechanisms underlying plant adaptation to environmental heterogeneity.

## Graphical abstract

This study investigated the stoichiometric traits and nutrient resorption efficiency of plant leaves across different elevational gradients on the northern slope of the Kunlun Mountains. The graphical abstract illustrates the geographic location of the study area and the vertical differentiation of plant communities. The results reveal patterns of variation in leaf nutrient traits along the elevation gradient, which are primarily influenced by soil nutrient availability, climatic conditions, and community composition.

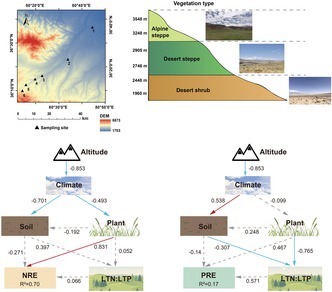

## 1 Introduction

Ecological stoichiometry studies the balance of chemical elements in organisms and their environments, offering a framework to integrate micro‐ and macro‐ecological theories. It provides critical insights into nutrient utilization strategies across trophic levels and evaluates nutrient limitations in ecosystems (Sterner and Elser 2002). Among the essential elements for plant growth, carbon (C), nitrogen (N), and phosphorus (P) serve distinct physiological roles, and their concentrations and ratios constitute the core of plant ecological stoichiometry (Elser et al. 2007; Lambers et al. 2008). Investigating the stoichiometric relationships among C, N, and P is thus fundamental to understanding biogeochemical cycles, productivity formation, and ecosystem stability‐maintain mechanisms (Vitousek et al. 2010).

Leaves, as primary photosynthetic organs, also reflect and determine plant growth status. Leaf stoichiometry and nutrient traits reveal nutrient availability and investment strategies under environmental variation (Wright et al. 2004), and help to infer nutrient limitation and adaptive strategies (Güsewell 2004). Latitudinal patterns in leaf stoichiometry have been widely documented at regional and global scales (Elser et al. 2010; Reich and Oleksyn 2004), but altitudinal patterns remain contentious. For instance, leaf N and P contents were found to first increase and then decrease with increasing elevation in the Mt. Gongga (Shi et al. 2012), whereas a study in the Altun Mountains reported the results of continuous increase (Zhang et al. 2021). These inconsistencies suggest ecosystem‐type‐specific patterns of C‐N‐P variation. Leaf N and P traits are also widely used to assess nutrient limitations. Especially, N:P ratio thresholds of 14/16 (Koerselman and Meuleman 1996) and 10/20 (Güsewell 2004) were commonly used to assess the nutrient limitation status, with the latter showing broader applicability in terrestrial ecosystems (Elser et al. 2010; Sardans et al. 2012). However, previous studies have shown that the 14/16 threshold hypothesis is more prone to distortion compared to the 10/20 threshold hypothesis (Yan et al. 2018).

Nutrient resorption is a key conservation strategy in plants, particularly under nutrient‐poor conditions (Yuan and Chen 2009, 2015). Generally, plants growing in harsh or infertile environments exhibit higher nutrient resorption efficiencies (NuRE), reducing dependence on soil nutrients (Xu et al. 2021; Yan et al. 2018). Understanding variation in NuRE can be helpful to explain plant nutrient strategies and nutrient cycling models (Brant and Chen 2015; Killingbeck 1996). Evaluating NuRE spatial patterns and driving factors also contributes to modeling nutrient fluxes and quantifying ecosystem productivity (Huang et al. 2023). NuRE altitudinal patterns are also debated, including increasing N resorption efficiency (NRE) (Bilgin and Güzel 2017; Gerdol et al. 2019) and reduced NRE trends (Tang et al. 2013; Zhang et al. 2015). Similarly, there is also no consensus on the dominant drivers of NuRE. Some studies emphasized the role of soil fertility (Brant and Chen 2015), while others highlighted the influence of climate in nutrient‐poor environments (Xu et al. 2021; Yan et al. 2018). Additionally, the relative limitation of N versus P can influence resorption efficiency, according to the relative resorption hypothesis. Plants tend to resorb more of the limiting nutrient prior to senescence (Güsewell 2004; Han et al. 2013). Stoichiometric homeostasis, a central concept in ecological stoichiometry, reflects an organism's physiological and biochemical regulation in response to environmental variability (Hessen et al. 2004; Koojiman 1995). The degree of homeostasis is highly correlated to adaptive capacity and ecological strategy (Frost et al. 2005), and at the community level, it often relates to ecosystem function and stability (Yu et al. 2010). In nutrient‐limited and variable terrestrial systems, high homeostasis may represent a conservative nutrient‐use strategy, and it is a key mechanism to maintain stability and competitive advantage (Elser et al. 2010; Yu et al. 2010).

Several hypotheses have been proposed to explain the main drivers of leaf stoichiometry and NuRE variation. The temperature‐biogeochemistry hypothesis suggests that temperature modulates soil microbial activity and nutrient mineralization, thereby altering leaf N and P contents (Reich and Oleksyn 2004). The temperature‐plant physiology hypothesis proposes that lower temperatures increase plant demand for N and P to compensate for reduced physiological efficiency. Although numerous studies have investigated the relationships between nutrient resorption and factors such as climate, vegetation type, and soil nutrient status (Yuan and Chen 2009; Yan et al. 2018), the key drivers regulating the plasticity of nutrient resorption remain unclear, and there is considerable heterogeneity in the environmental responses of NRE and PRE (Han et al. 2011). Most studies suggest that plant nutrient resorption strategies are regulated by temperature and precipitation, with PRE being particularly sensitive to climatic factors (Chen et al. 2013; Xu et al. 2021). In addition, some research has indicated that nutrient resorption efficiency may also be significantly influenced by leaf functional traits (Achat et al. 2018; Mediavilla and Escudero 2003). Therefore, identifying the main drivers of nutrient resorption is crucial for understanding the adaptive mechanisms of plants. Altitudinal gradients, with their steep climatic variation over short distances, offer a natural laboratory for testing these ecological hypotheses (Körner 2007). The northern slope of the Kunlun Mountains encompasses semi‐shrub desert, desert steppe, and alpine steppe (Zhang et al. 2025), making it an ideal system for examining plant nutrient strategies under environmental gradients. In this study, we investigated leaf and soil nutrient contents across elevations ranging from 1960 to 3548 m. Our objectives were to: (1) characterize the variation of community leaf C–N–P stoichiometry along the elevational gradient; (2) assess nutrient limitation types and stoichiometric homeostasis; and (3) identify key environmental factors and their pathways influencing N:P ratios and NuRE. We hypothesize that: (1) influenced by the high spatial heterogeneity of desert ecosystems and the pronounced climatic gradients in mountainous regions, the leaf stoichiometric traits and nutrient resorption efficiency of plant communities are expected to exhibit non‐linear variation patterns along the elevational gradient; (2) both community N and P maintain homeostasis across the gradient; and (3) during plant growth, N and P perform distinct physiological functions, thus NRE and PRE exhibit differential responses to environmental factors. This study aims to enrich stoichiometric theory and provide insights for vegetation conservation and restoration on the northern slope of the Kunlun Mountains.

## 2 Materials and Methods

### 2.1 Study Area and Sample Collection

**FIGURE 1:**
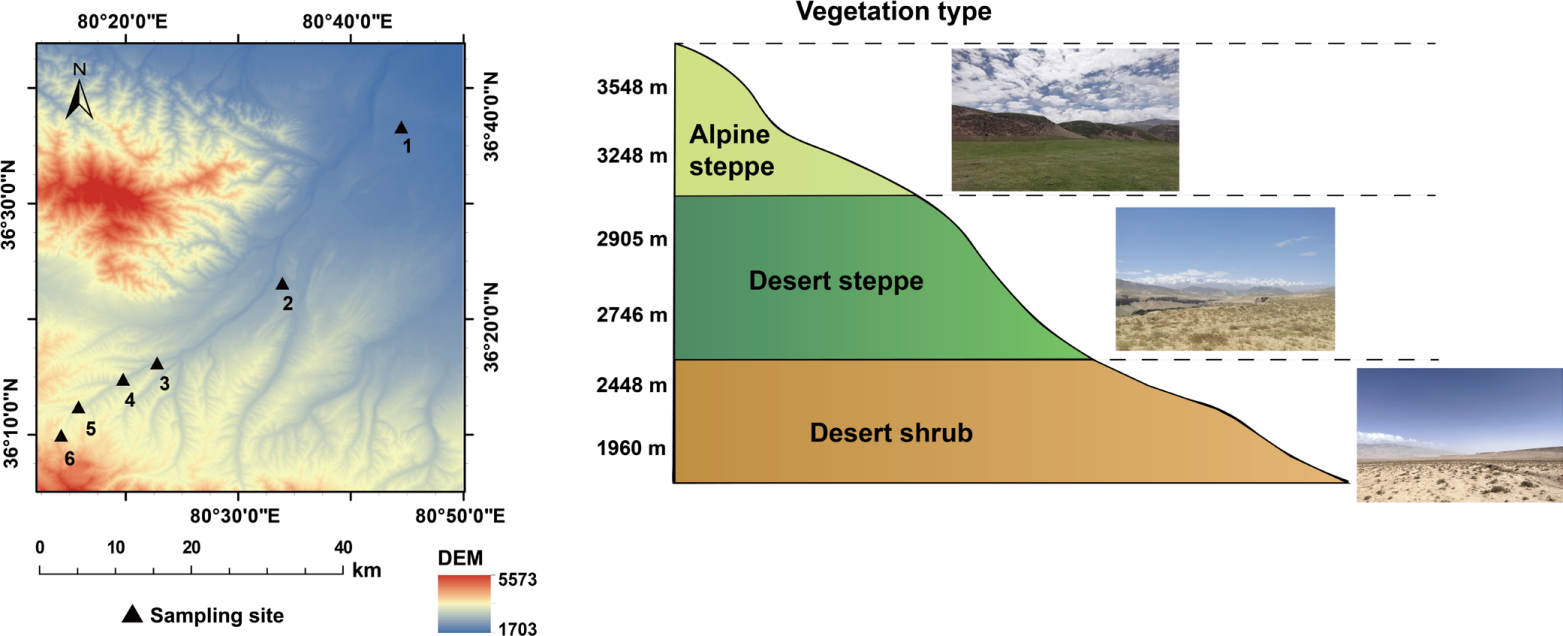
Location of the study area and representative plots along the elevational gradient on the northern slope of Mt. Middle Kunlun.

The northern slopes of the Kunlun Mountain chain are located along the northern edge of the Qinghai‐Tibet Plateau (36°12′13.16″–36°46′48.82″ N, 80°15′46.56″–80°57′59.49″ E). Here, the climate is controlled by the Mongolian‐Siberian anticyclone, and conditions are harsh. The middle of the Kunlun Mountain chain stretches between 77°24′ and 84° E and is extremely arid. Vegetation is scarce, and plant species richness is generally low. In this study, six sampling sites were selected along the elevation gradient ranging from 1960 to 3548 m, with semi‐shrub desert (1960 and 2448 m), desert steppe (2746 and 2905 m) and alpine steppe (3248 and 3548 m) (Figure 1). The data for the mean annual temperature (MAT) and mean annual precipitation (MAP) at each elevation gradient were obtained from meteorological stations established at the sampling sites (HOBO U30, Onset Computer Corporation, USA). The device continuously records on‐site meteorological data over the long term, ensuring an accurate representation of environmental conditions across different elevations. Detailed information on the study area, including elevation gradients, vegetation types, MAT, and MAP, is provided in (Table S1).

In mid‐July 2022, vegetation surveys and sample collection were conducted along six distinct elevation gradients. At elevations of 1960 m and 2448 m, four 10 m × 10 m shrub plots were established at each site, with approximately 100 m spacing between plots. For sites between 2746 m and 3548 m, four 1 m × 1 m herbaceous plots were set up at each sampling point, with approximately 10 m spacing. In the shrub and herbaceous plots, species name, plant height, density and canopy size were recorded, above‐ground samples were collected for dry biomass estimation in each community, and then mature and healthy leaves were collected and sorted by species for dry biomass estimation and chemical analysis.

Litter samples were collected in October during the natural senescence period to assess nutrient resorption characteristics. In the shrub communities (1960–2448 m), four 1 m × 1 m litter traps were set up for each shrub species, dead leaves were gently shaken from the shrubs, and a plastic net was placed on the ground to collect naturally fallen leaves; then these samples were mixed by species and preserved for subsequent nutrient analysis. In herbaceous communities (2746–3548 m), within each 1 m × 1 m plot, litter samples were all collected and not classified due to the difficulty in identifying species from the senesced herbaceous plants. All plant samples were immediately placed in kraft paper bags and transported to the laboratory, where they were dried at 65°C to a constant weight. The samples were then ground and sieved for analysis of C, N, and P concentrations as well as their stoichiometric characteristics.

After removing surface litter and humus layers, soil samples were collected along diagonal lines within each plot. Five soil samples were taken randomly from the 0 to 30 cm depth at each sampling point using a soil auger and mixed to form a composite sample. A total of 24 soil samples were collected across the six elevation gradients. All soil samples were air‐dried in the laboratory, passed through a 2 mm sieve, and stored for subsequent analysis of soil physical and chemical properties.

### 2.2 Plant and Soil Physicochemical Measurements

Plant carbon and nitrogen contents were determined using a C/H/N elemental analyzer (Vario El III, Hanau, Germany). Plant phosphorus content was measured using an inductively coupled plasma emission spectrometer (ICAP 6300, Waltham, USA). Soil physicochemical properties, including total nitrogen (TN), total phosphorus (TP), ammonium nitrogen (AN), available phosphorus (AP), soil water content (SWC), pH, and electrical conductivity (EC), were measured following the methods outlined by Zhang et al. (2024). After chemically dispersing soil aggregates, the particle size distribution of particulate organic carbon (POC) and mineral‐associated organic carbon (MAOC) was determined as described by Cotrufo et al. (2019). MAOC was obtained by dispersing the soil in 50 mL of sodium hexametaphosphate and passing it through a 53 μm sieve. Organic carbon in the 53–2000 μm fraction was designated as POC. Soil organic carbon (SOC) was calculated as the sum of MAOC and POC content. Total salt content (TS) was calculated by summing the concentrations of eight ions: CO₃^2−^, HCO₃^−^, Cl^−^, SO₄^2−^, Ca^2+^, Mg^2+^, Na^+^ and K^+^, as measured by ion chromatography (Bradfield and Cooke 1985).

### 2.3 Calculation of Plant Community Traits

#### 2.3.1 Community Weighted Mean (CWM)

In this study, CWM of functional traits was used to represent functional composition. This index quantifies the weighted values of different traits in the community based on the species biomass, effectively reflecting the mass‐ratio effect (Hao et al. 2020; Violle et al. 2007). The calculation formula is as follows:

$${\mathrm{CWM}}_{t}=\sum_{i=1}^{N}t_{i}\times w_{i}$$

where CWM_*t*_ is the community weighted mean for trait *t*, *t*_*i*_ is the trait value for species *i*, and *w*_*i*_ is the biomass proportion of species *i* in the community. Thus, in this study, we used this formula to calculate the total C, N, and P content in plant community leaves (Zhang et al. 2023). The formulas are as follows:

$$\mathrm{LTC}=\sum_{i=1}^{s}B_{i}C_{i}$$

$$\mathrm{LTN}=\sum_{i=1}^{s}B_{i}N_{i}$$

$$\mathrm{LTP}=\sum_{i=1}^{s}B_{i}P_{i}$$

where LTC, LTN, and LTP represent the total C, N, and P content in the leaves of the community, respectively, B_*i*_ is the relative biomass of species *i*, C_*i*_, N_*i*_, and P_*i*_ are the total C, N, and P content in the leaves of species *i*, respectively.

#### 2.3.2 Shannon–Wiener Index (H′) and Pielou's Evenness Index (E)

The Shannon–Wiener diversity index and Pielou's evenness index are used to represent the α‐diversity of the plant community (Magurran 1988).

$$H^{'}=-\sum_{i=1}^{S}P_{i}\ln P_{i}$$

$$E=H^{'}/\mathrm{lnS}$$

where P_*i*_ = *n*_*i*_/*N*, *n*_*i*_ represents the number of individuals of the *i* species, *N* is the total number of individuals, and *S* is the number of species in the plot.

#### 2.3.3 Nutrient Resorption Efficiency (NuRE) and Its Control Strategies

NuRE is defined as the percentage of nutrients resorbed from senescing leaves. The calculation formula is as follows:

$$\text{NuRE}=\left(1-{\text{Nutrient}}_{\text{senesced}}/\text{Nutrien}t_{\text{green}}\times \text{MLCF}\right)\times 100\%$$

where Nutrient_senesced_ and Nutrient_green_ are the nutrient concentrations in senescing and green leaves, respectively. The mass loss correction factor (MLCF) is used to correct the estimation bias in resorption efficiency based on leaf senescence, as proposed by Vergutz et al. (2012). In this study, an MLCF value of 0.762 was used.

The nutrient resorption control strategy of plant communities was assessed following the approach of Sun et al. (2023). Specifically, to evaluate the presence of nutrient limitation control, a power‐law model was established to characterize the relationship between LTN:LTP and ${\text{Resorbed}}_{\mathrm{LTN}:\mathrm{LTP}}:{\text{Resorbed}}_{\mathrm{LTN}:\mathrm{LTP}}=\omega \times {\left(\mathrm{LTN}:\mathrm{LTP}\right)}^{\lambda }$.

where *ω* and *λ* are the regression parameters. LTN:LTP represents the nutrient concentration ratio in green leaves, and Resorbed_LTN:LTP_ denotes the absolute ratio of resorbed nutrients from senesced leaves, calculated as:

$${\text{Resorbed}}_{\mathrm{LTN}:\mathrm{LTP}}=\frac{\mathrm{LTN}-{\mathrm{LTN}}_{S}\times \text{MLCF}}{\mathrm{LTP}-{\mathrm{LTP}}_{S}\times \text{MLCF}}$$

When *λ* < 1 or *λ* > 1, nutrient resorption is interpreted to follow a nutrient limitation control strategy. When *λ* = 1, it indicates a stoichiometric control strategy.

In addition, to more directly assess the presence of stoichiometric control, a linear regression was performed between LTN:LTP and Resorbed_LTN:LTP_. If a positive correlation exists and the regression slope does not significantly differ from 1, it indicates that the resorption of these two nutrients follows stoichiometric control. Conversely, if the slope significantly deviates from 1, it suggests that nutrient resorption in the plant community is jointly regulated by both nutrient limitation and stoichiometric control strategies.

#### 2.3.4 Leaf Stoichiometric Homeostasis

The response of plant nutrient stoichiometry to environmental variation across different growth stages can be quantified using the homeostasis index. The homeostasis coefficient *H* was calculated using the following equation:

$$\mathrm{lg}\left(y\right)=\mathrm{lg}\left(c\right)+\mathrm{lg}\left(x\right)/H$$

where *H* is the homeostasis index; *c* is a constant; *x* is the soil stoichiometry; and *y* is the stoichiometry of the N:P ratio of dominant shrubs. Following Persson et al. (2010), when the regression equation was significant (*p* < 0.05), values of *H* ≥ 4 were classified as a steady state, 2 ≤ *H* < 4 as a weak steady state, 1.33 ≤ *H* < 2 as a weak sensitive state, and *H* < 1.33 as a sensitive state. However, when the regression equation fit was not significant (*p* > 0.05), the case in question was considered an absolute steady state.

### 2.4 Statistical Analyses

To investigate the variation patterns of community‐level leaf nutrient concentrations, NuRE, and soil physicochemical properties along the elevational gradient, first‐, second‐, and third‐order polynomial regression models were fitted, and the best‐performing model was selected for presentation. Pearson correlation analysis was conducted to examine the relationships between community leaf nutrient traits and environmental factors, including soil and climatic variables. Random forest analysis was employed to assess the relative importance of these environmental predictors. To identify the key factors influencing community nutrient limitation and NuRE, a random forest model was constructed using standardized environmental variables, and variables with high importance scores were further selected for multiple linear regression analysis to quantify their relative contributions to plant nutrient traits. To further elucidate the regulatory pathways of ecosystem components on NuRE, a partial least squares path model (PLS‐PM) was constructed. Prior to modeling, collinearity among predictors was assessed using both random forest variable rankings and variance inflation factor (VIF) analysis, and highly collinear variables were excluded to enhance model stability and interpretability. The model was then used to estimate the direct and indirect effects of latent variables on NuRE. The statistical analyses and data visualizations above were conducted in R software (v4.3.2, R Core Team 2024) using the packages tidyverse, pheatmap, rfPermute, MuMIn, glmm.hp., relaimpo, plspm, and ggplot2. In addition, 95% confidence intervals were used to assess whether the regression slopes significantly differed from 1 when evaluating nutrient resorption control strategies. This analysis was performed using SPSS 27 (SPSS Inc.).

## 3 Results

### 3.1 Variations in Soil Physicochemical Properties and Leaf Stoichiometry Along the Elevational Gradient

**FIGURE 2:**
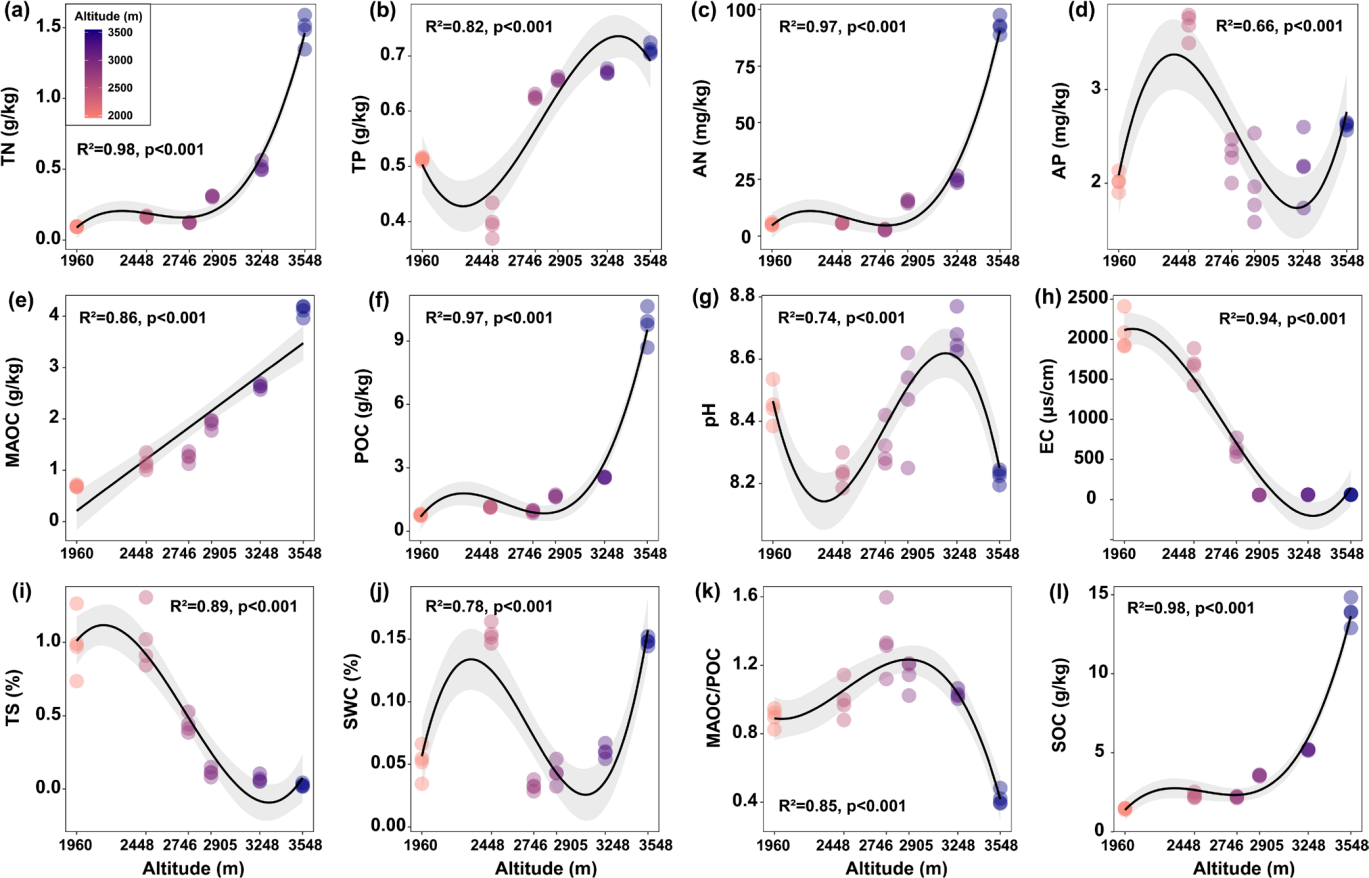
First‐, second‐, and third‐order polynomial regressions were used to fit the variation trends of soil physicochemical properties along the elevational gradient in order to evaluate the fitting performance of different models and their ecological implications.

**FIGURE 3:**
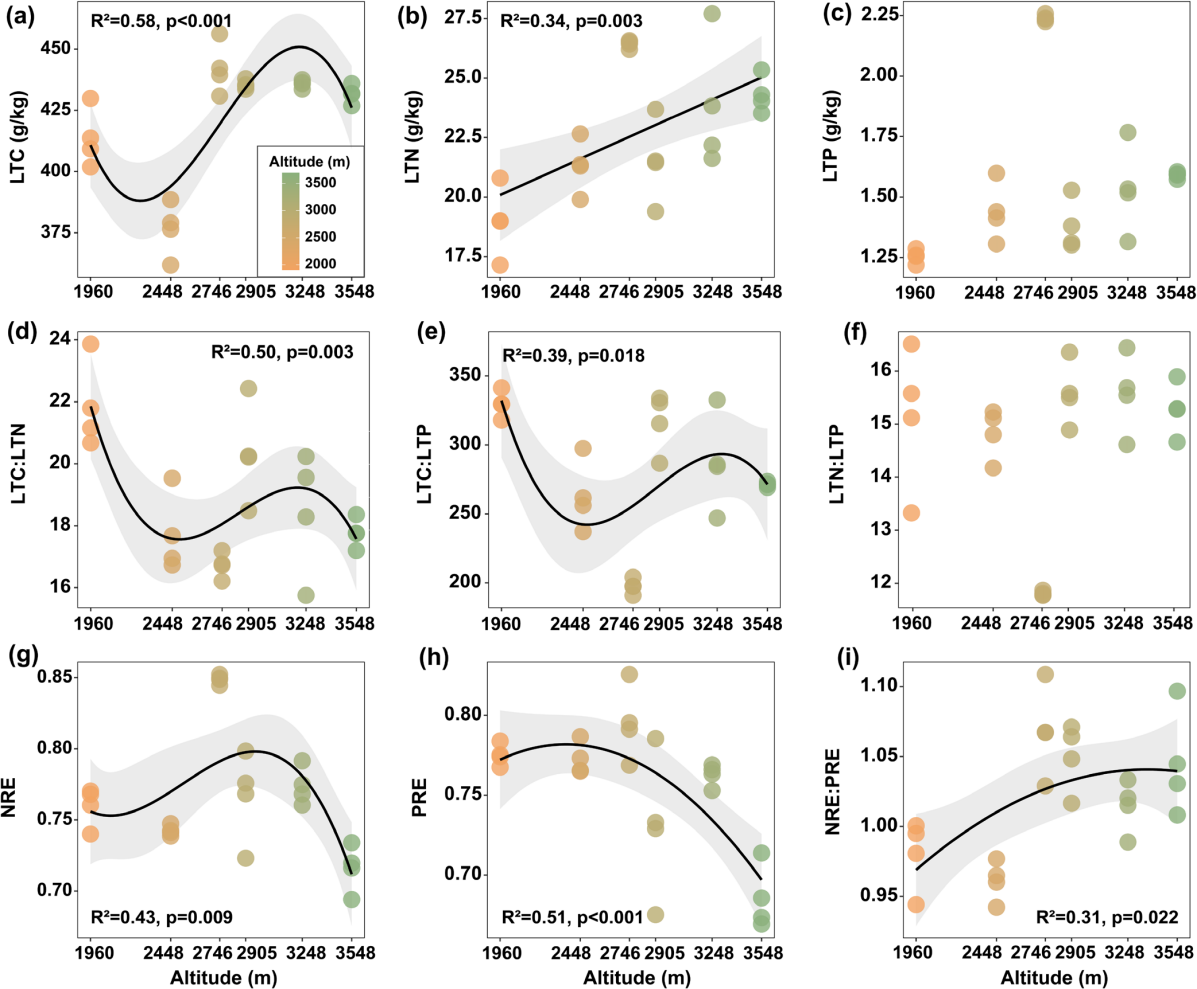
First‐, second‐, and third‐order polynomial regressions were used to fit the variation trends of plant community nutrient concentrations and their stoichiometric ratios along the elevational gradient.

**FIGURE 4:**
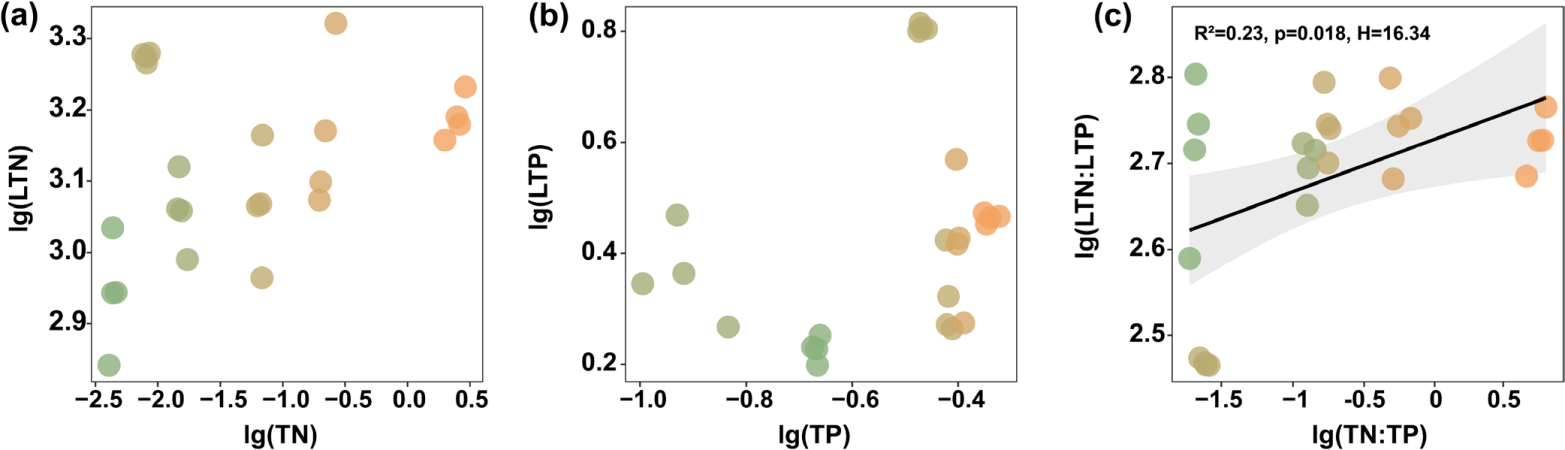
Associations between community‐level leaf nutrient concentrations and total soil nutrient contents, with all variables log‐transformed before statistical analysis.

Soil TN, AN, MAOC, POC, and SOC exhibited significant increases with increasing elevation (Figure 2a,c,e,f,l), whereas TS and EC showed significant decreases (Figure 2h,i). TP showed a pattern of first decreasing, then increasing, and finally decreasing again, whereas AP exhibited the opposite trend, first increasing, then decreasing, and finally increasing again (Figure 2b,d). The MAOC:POC ratio peaked at 2905 m (Figure 2k). LTC:LTN and LTC:LTP ratios followed significant hump‐shaped patterns along the elevational gradient (*p* < 0.01; Figure 3d,e), while LTN increased linearly with increasing elevation (*p* = 0.003, Figure 3b). No significant trend was observed for LTP or LTN:LTP ratio (Figure 3f). NRE exhibited a significant hump‐shaped trend with elevation (Figure 3g), whereas PRE and the NRE:PRE ratio followed inverse U‐shaped trends (Figure 3h,i). Community coverage, aboveground biomass, and the Shannon diversity index all increased linearly with elevation, whereas the Pielou evenness index showed no significant change (Figure S1). LTN and LTP did not exhibit significant trends with increasing soil TN or TP, indicating strict homeostasis (*p* > 0.05). The LTN:LTP ratio increased linearly with soil TN:TP, exhibiting homeostatic behavior based on fitted model (*p* < 0.05, *H* = 16.34, Figure 4c).

### 3.2 Control Strategies of Community‐Level Leaf Nutrient Resorption

**FIGURE 5:**
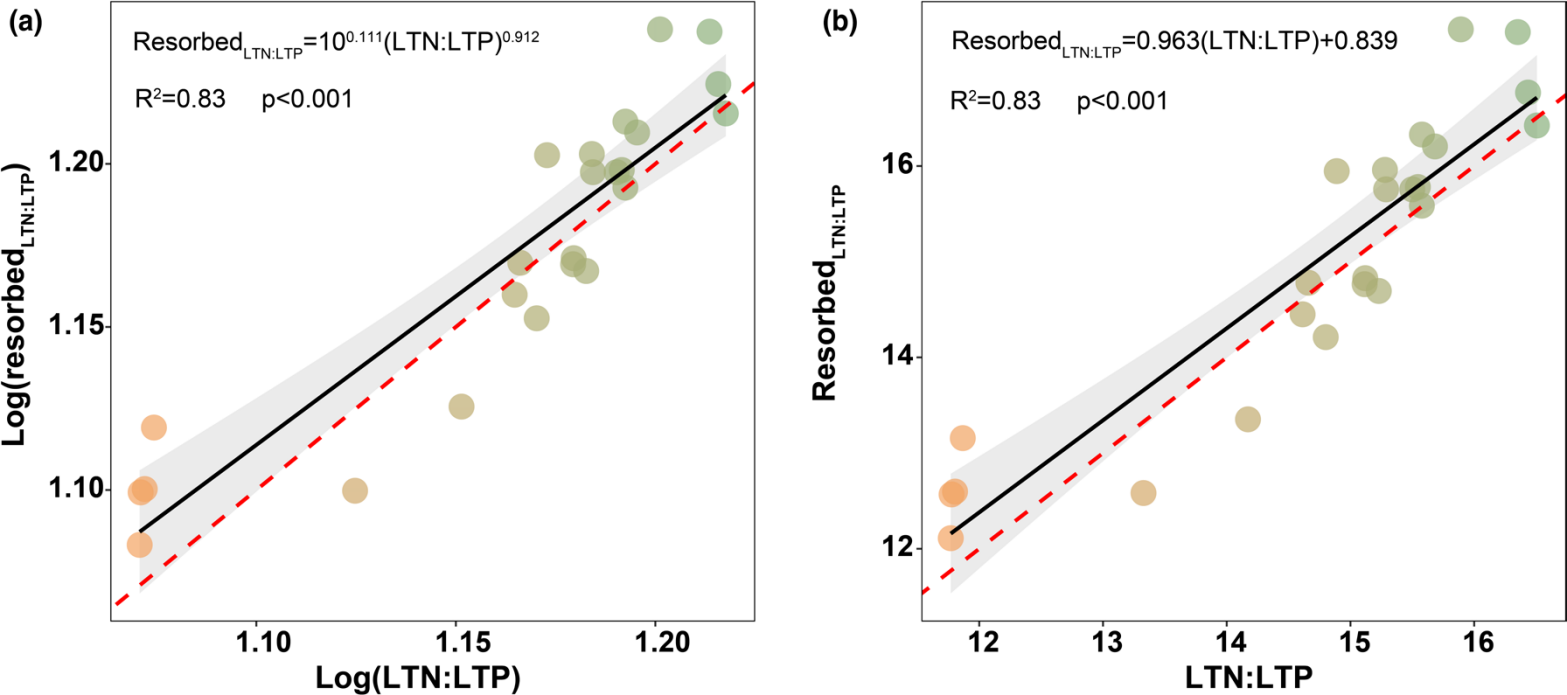
Power‐law regression (a) and linear regression (b) between resorbed LTN:LTP and LTN:LTP. A red dashed line indicating the 1:1 ratio is included as a reference.

The regression slope (*λ*) between Log(resorbed LTN:LTP) and Log(LTN:LTP) was 0.912, which did not significantly differ from 1 (Figure 5a), indicating that nutrient resorption followed stoichiometric control. Similarly, the slope between resorbed LTN:LTP and LTN:LTP was 0.963, also not significantly different from 1 (Figure 5b), further suggesting that the N and P resorption processes of plant communities in the study region were primarily governed by stoichiometric control.

### 3.3 Responses of Community‐Level Leaf Nutrient Resorption to Environmental Factors

**FIGURE 6:**
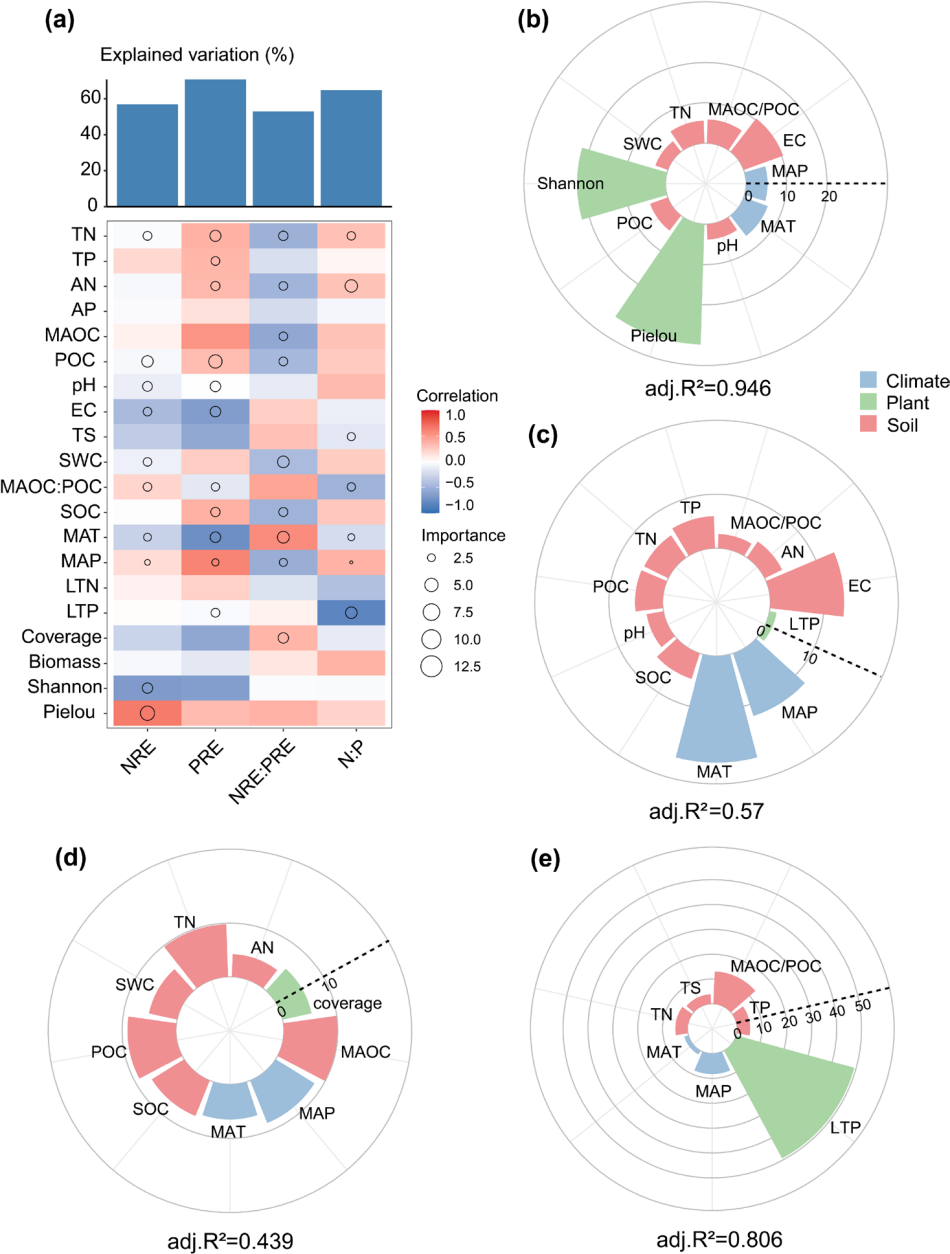
Spearman correlation and random forest analyses were used to examine the relationships between biotic and abiotic factors and plant nutrient characteristics along the elevational gradient. Figure (a) shows the Spearman correlation and the total explanatory power of environmental factors derived from random forest analysis. Figures (b) and (c) display the responses of NRE and PRE to the selected predictors, while Figures (d) and (e) show the responses of NRE:PRE and LTN:LTP to multiple predictors. Random forest was first applied to identify the relatively important predictors, followed by multiple regression models to determine the relative importance of each factor. *R* ^2^ values indicate the proportion of variance explained by the models.

Correlation and random forest analyses revealed that the total variation in the response variables explained by biotic and abiotic factors followed the order in PRE > LTN:LTP > NRE > NRE:PRE. The random forest results identified TN, POC, pH, EC, SWC, SOC, MAT, MAP, and the Shannon and Pielou indices as key predictors of NRE. TN, TP, AN, POC, pH, EC, MAOC:POC, SOC, MAT, MAP, and LTP were identified as important indicators for predicting PRE. TN, TP, MAOC, POC, SWC, SOC, MAT, MAP, and vegetation coverage influenced the NRE:PRE ratio, while TN, TP, TS, MAOC:POC, MAT, MAP, and LTP were crucial for predicting the LTN:LTP (Figure 6a). Multiple linear regression models constructed with key variables identified by the random forest approach explained 94.6% of the variation in NRE, 57.0% in PRE, and 80.6% in LTN:LTP. However, these models accounted for only 43.9% of the variation in the NRE:PRE ratio (Figure 6b–e), suggesting that the NRE:PRE ratio is regulated by a more complex interplay of factors that cannot be fully explained by environmental variables alone. Shannon and Pielou indices had relatively strong effects on NRE, whereas EC, MAT, and MAP had greater impacts on PRE. LTN:LTP was primarily influenced by LTP.

**FIGURE 7:**
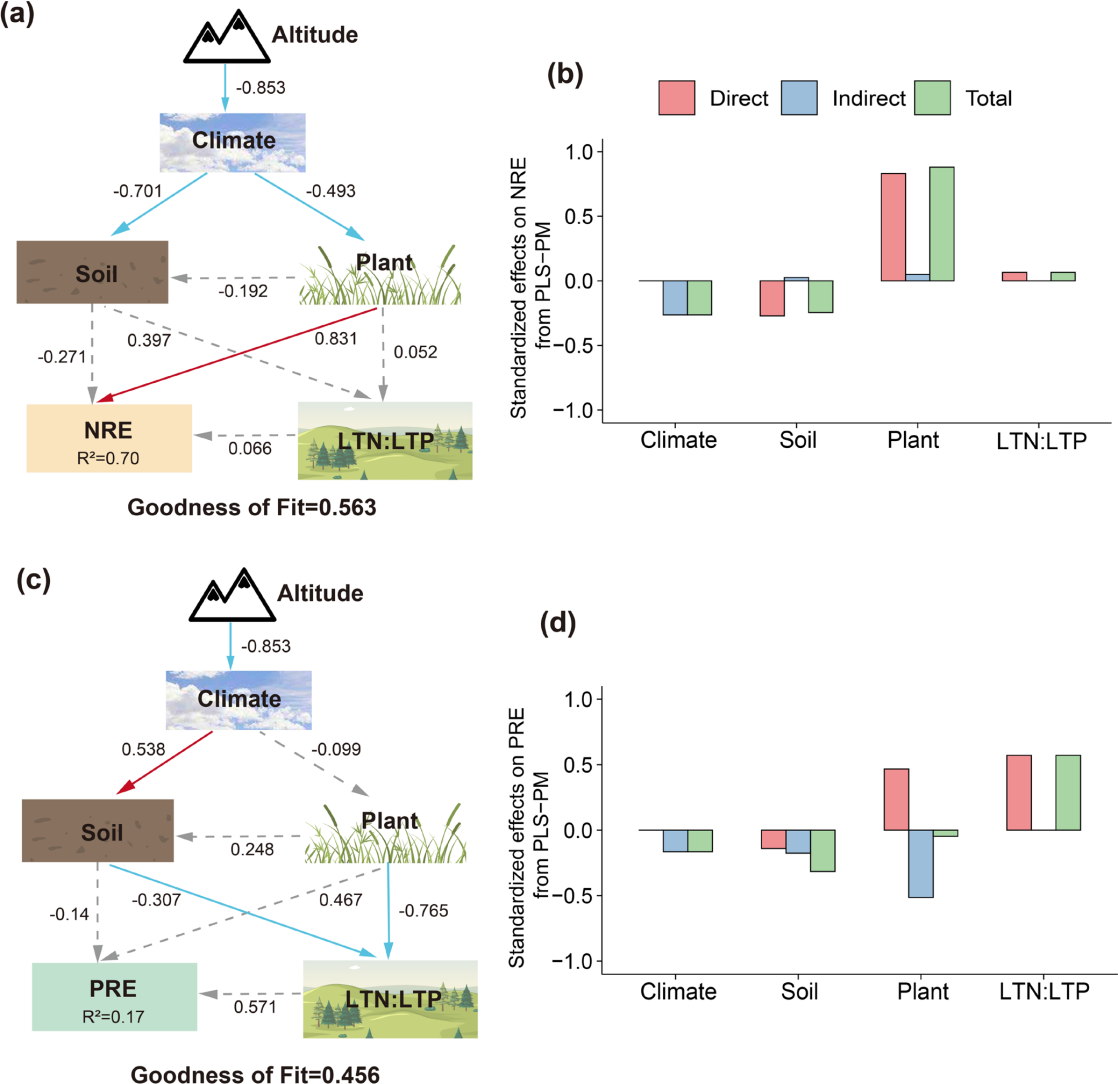
Figure (a) and (c) present PLS‐PM illustrating the effects of biotic and abiotic factors on NRE, PRE, and LTN:LTP. Significant variables identified by the random forest analysis were first tested for multicollinearity, and only those that passed were included in the final models. Red and blue solid arrows indicate significant positive and negative causal relationships (*p* < 0.05), respectively, while gray arrows represent non‐significant paths. *R* ^2^ values denote the proportion of variance in the response variables explained by the models. Numbers adjacent to arrows represent standardized path coefficients. Figure (b) and (d) show the direct, indirect, and total effects of each factor on NRE and PRE, respectively.

To further explore the regulatory pathways of biotic and abiotic drivers on nutrient resorption, we constructed PLS‐PM under the assumption that variation in NRE and PRE is driven by elevation, climate, community structure, and soil properties. The two models yielded goodness‐of‐fit (GoF) values of 0.563 and 0.456, respectively (Figure 7a,c), indicating moderate explanatory power under complex ecological conditions. Environmental variables explained 70% of the variation in NRE and 17% in PRE (Figure 7a,c). Notably, plant characteristics exerted strong direct effects on NRE but weak direct effects on PRE, while LTN:LTP had a stronger direct influence on PRE than on NRE (Figure 7b,d). Climate factors indirectly influenced NRE through their effects on plant traits but showed no significant indirect effect on PRE via plants or soil (Figure 7a,c).

## 4 Discussion

### 4.1 Variation in Leaf Stoichiometry and NuRE Along the Elevation Gradient

Previous studies have shown that the variation in plant community nutrient contents differs among ecosystems (Reich and Oleksyn 2004). In our study area, the vegetation type at elevations of 1960–2448 m is classified as montane semi‐shrub desert, where the LTC, LTN, and LTP concentrations range from 434.8 to 456.155 g/kg, 23.5–27.7 g/kg, and 1.59–2.26 g/kg, respectively. These values are notably higher than the global and national averages for shrublands (N: 19.1 g/kg; P: 1.11 g/kg) (Han et al. 2005). The northern slope of Mt. Kunlun is located near the Tarim Basin and is significantly influenced by dust transport from the westerlies. Previous studies have shown that long‐term desert dust deposition markedly enhances soil nutrient levels and has a positive impact on plant growth (Wang et al. 2021).

Numerous studies have reported nonlinear relationships between leaf stoichiometric traits and elevation (Shi et al. 2012; Fisher et al. 2013; Zhao et al. 2014). Consistent with these findings, we observed non‐linear patterns in LTC, LTC:LTN, and LTC:LTP. Notably, LTN content increased with elevation, in agreement with previous studies, suggesting that in high‐elevation and low‐temperature environments, plants may enhance intracellular metabolic processes by increasing their N and P concentrations (Zhang et al. 2021; Sanaullah et al. 2014). According to the Temperature‐Plant Physiology Hypothesis, plant metabolic rates are temperature‐dependent, and under low‐temperature conditions, plants require higher N and P contents to compensate for reduced physiological efficiency (Reich and Oleksyn 2004). However, in our study, LTP content did not exhibit a similar increasing trend with elevation. Considering the concurrent decline in soil AP at higher elevations (Figure 2d), we speculate that this pattern may be due to intensified weathering processes at high altitudes. These processes facilitate the transformation of primary mineral phosphorus into secondary forms (Lajtha and Schlesinger 1988), such as calcium‐bound phosphate, which is less bioavailable to plants, ultimately leading to reduced uptake of available P. A global meta‐analysis reported mean NuRE of 48.4% for nitrogen and 53.3% for phosphorus (Yan et al. 2018), both significantly lower than the NuRE values observed in this study (71.6%–85.2% for NRE; 66.9%–82.6% for PRE). This indicates that under nutrient‐poor conditions, plants enhance nutrient conservation by maintaining high resorption efficiency (Xu et al. 2021). In our study, both LTN and NRE increased with elevation, ranging from lower to mid elevations (1960–2905 m), suggesting a high investment–high return N‐use strategy in response to an infertile soil environment. However, at higher elevations (3248 and 3548 m), NRE dropped sharply. This may be due to increased soil TN and AN (Figure 2), reducing plant reliance on internal N recycling. Previous studies have suggested that both LTN:LTP and NRE:PRE can serve as effective indicators of plant nutrient limitation status (Du et al. 2020; Güsewell 2004; Han et al. 2013). In this study, LTN:LTP ranged from 11.91 to 16.39, indicating a state of co‐limitation by N and P according to the 10/20 threshold theory (Güsewell 2004). Such co‐limitation is common in nutrient‐poor environments (Du et al. 2020). The NRE:PRE ratio increased continuously along the elevational gradient, suggesting an intensification of N limitation with increasing elevation. The ratio tended to stabilize at higher elevations, indicating that the resorption of N and P became more balanced. This pattern suggests a minimal difference in the availability of N and P in the soil at high elevations, reducing the necessity for plants to preferentially resorb one element over the other. Stoichiometric homeostasis, which represents an organism's capacity to regulate internal nutrient composition despite environmental fluctuations, was evident in LTN, LTP, and LTN:LTP values. This suggests that across the elevation gradient, species adopt conservative nutrient strategies to ensure persistence under stress (Elser et al. 2010; Yu et al. 2010).

### 4.2 Control Strategies of Community‐Level Leaf Nutrient Resorption

Previous studies have shown that stoichiometric control tends to operate at the community level, whereas nutrient limitation control is more species‐specific (Chen et al. 2021), which is consistent with our findings. Our results indicate that leaf nutrient resorption in desert plants is predominantly governed by stoichiometric control at the community scale. Similar patterns have been reported in epiphytic plant communities, where stoichiometric control strategies are more prevalent than nutrient limitation strategies, and this trend has also been confirmed in terrestrial plants (Chen et al. 2021; Sun et al. 2023). For most nutrient elements, stoichiometric control is more widespread and plays a critical role in maintaining elemental balance within plants (Sun et al. 2023).

In desert ecosystems, where resources are scarce and environmental stress is intense, plants face high costs in acquiring energy and utilizing nutrients. As a regulation strategy that relies on osmotic gradients between sources and sinks and requires relatively low energy input, stoichiometric control is likely to confer greater adaptive advantages under such conditions (Chapin and Kedrowski 1983). Based on the analysis of LTN:LTP, our study found that plants across the elevational gradient in the study area generally exhibit co‐limitation by N and P, with no evidence of element‐specific nutrient limitations. Under these circumstances, plants are more likely to benefit from adopting stoichiometric control mechanisms to optimize nutrient resorption, thereby achieving greater efficiency in energy and resource allocation.

### 4.3 Responses of Plant Nutrient Limitation and NuRE to Biotic and Abiotic Factors

The research showed that the NRE:PRE ratio was mainly influenced by soil physicochemical properties, while LTN:LTP was primarily driven by foliar P concentrations (Figure 6d,e), which is consistent with previous findings (Güsewell and Koerselman 2002; Ordoñez et al. 2009). These results implied that LTN:LTP may reflect long‐term adaptation to environmental nutrient availability, while NRE:PRE may respond dynamically to short‐term changes, such as short‐term shifts in the availability of soil nutrients. Therefore, the combined application of the LTN:LTP and the NRE:PRE ratios in assessing nutrient limitation can facilitate a more comprehensive understanding of plant nutrient‐use strategies in resource‐limited ecosystems.

Correlation analyses and random forest modeling identified soil TP and TS as primary drivers of LTC variation along the elevation gradient (Figure S2a,b). This may reflect osmotic adjustments in response to salinity, whereby plants synthesize carbon‐rich osmolytes such as proline, soluble sugars, and non‐structural carbon concentrations in leaves (Parida and Das 2005). In contrast, soil N and P were the main predictors of LTN (Figure S2a,c). This aligned with previous findings that soil available N is a key determinant factor of foliar N content (Reich and Oleksyn 2004), while P also enhances N uptake by facilitating metabolic processes (Townsend et al. 2007; Vitousek et al. 2010; Zhang et al. 2015).

Leave traits associated with P are generally more sensitive to environmental variation than those associated with N (Han et al. 2011). Several studies have shown that changes in NRE are more driven by plant traits, whereas PRE is more strongly influenced by climate and soil conditions (Chen et al. 2013; Xu et al. 2021; Yan et al. 2018). This asymmetry may originate from the differences in biogeochemical cycling—N is abundant in the atmosphere and benefits from biological fixation, while P primarily derives from parent material and is less mobile in soil (Aerts and Chapin 1999; Chen et al. 2013). This study also yielded a similar conclusion: NRE was significantly influenced by plant community diversity, whereas PRE was primarily driven by climatic factors (Figure 7). This divergence may also help explain why NRE did not exhibit a linear trend along the elevational gradient. Existing theories suggest that in cold regions, NRE in leaves is generally higher than PRE. Specifically, PRE tends to be greater than NRE in low‐elevation areas, such as tropical, subtropical, and temperate regions, whereas in high‐elevation and high‐latitude regions, NRE surpasses PRE (Du et al. 2020; Hou et al. 2021; Yang et al. 2025). Similarly, our study observed a significant decrease in PRE with increasing elevation. This pattern may be attributed to several factors. First, intensified weathering processes at high elevations may increase the availability of soil P, thereby reducing plant dependence on internal P recycling. Second, low temperatures at high elevations can suppress physiological activities during leaf senescence, particularly the metabolic processes and enzymatic functions involved in P translocation, ultimately hindering efficient P resorption (Xu et al. 2022). Additionally, low‐elevation areas generally receive less precipitation and are often associated with nutrient‐poor soils. Under such conditions, plants may adjust their nutrient conservation strategies to cope with resource limitations, typically by enhancing nutrient resorption efficiency to reduce nutrient loss (Xu et al. 2021; Zhu et al. 2025).

Previous studies often attributed variations in nutrient resorption to absolute nutrient availability; however, nutrient resorption is also regulated by relative nutrient limitation (Han et al. 2013). Our PLS‐PM results confirmed that plant traits significantly influence NRE. However, LTN:LTP exerted only a weak effect on NRE, while it strongly affected PRE, which supported the conclusion that PRE is more tightly linked to stoichiometric imbalance and relative nutrient status (Han et al. 2013). Notably, environmental variables explained only 17% of PRE variation (Figure 7b), suggesting that PRE may be governed more by phylogenetic constraints and plant life form than by external conditions.

## 5 Conclusions

Plant communities across different elevations in the study area were co‐limited by N and P, with N limitation intensifying as elevation increased. Both NRE and PRE were higher than the global average, reflecting adaptive strategies developed by plants in nutrient‐poor environments. At high elevations, plant communities showed a significantly reduced dependence on soil nutrients and a relatively balanced resorption of N and P. Soil TP and TS were the primary drivers of LTC variation, while LTN was mainly influenced by soil N and P availability. NRE was significantly affected by plant community diversity, whereas PRE was primarily driven by climatic factors. Furthermore, we found that nutrient resorption processes across different elevations are regulated by stoichiometric control strategies, which enable plant communities to optimize their capacity and resource allocation under nutrient‐poor conditions. Additionally, LTN:LTP had a significantly stronger influence on PRE than on NRE, indicating that PRE is more sensitive to relative nutrient status and stoichiometric imbalance. We propose that the NRE:PRE ratio can serve as a dynamic indicator responding to short‐term environmental changes, while LTN:LTP reflects plants' adaptive strategies to long‐term nutrient limitations. The combined use of these two indices facilitates a more comprehensive and accurate assessment of nutrient limitation status in plant communities. This study enhances our understanding of nutrient limitation differentiation and adaptive mechanisms of plant communities along elevational gradients in arid mountain ecosystems.

## Author Contributions

**Shuwen Xue:** formal analysis (equal), methodology (equal), visualization (equal), writing – original draft (equal). **Atawula Jiashalaiti:** conceptualization (equal), data curation (equal), investigation (equal). **Dongdong Zhang:** investigation (equal), methodology (equal). **Zhihao Zhang:** investigation (equal), methodology (equal), resources (equal). **Lei Li:** investigation (equal), methodology (equal). **Bo Zhang:** investigation (equal), methodology (equal). **Fanjiang Zeng:** resources (equal). **Yan Lu:** conceptualization (equal), funding acquisition (equal), project administration (equal), resources (equal), supervision (equal).

## Conflicts of Interest

The authors declare no conflicts of interest.

## Supporting information


**Data S1:** ece372083‐sup‐0001‐Supinfo.docx.

## Data Availability

All data used in the study are included in this paper and are available.
