# Supplementary material for: Variation in Community Leaf Stoichiometry and Nutrient Resorption Along an Elevational Gradient on the Northern Slope of the Kunlun Mountains
Source: Ecol Evol. 2025 Aug 29;15(9):e72083. doi: 10.1002/ece3.72083 (PMC12395183; doi:10.1002/ece3.72083)
Supplement: Supplementary file 1 — Data S1: ece372083‐sup‐0001‐Supinfo.docx. [file ECE3-15-e72083-s001.docx]

Fig.S1 Linear relationships between plant community coverage, aboveground biomass, and α-diversity across different elevations.


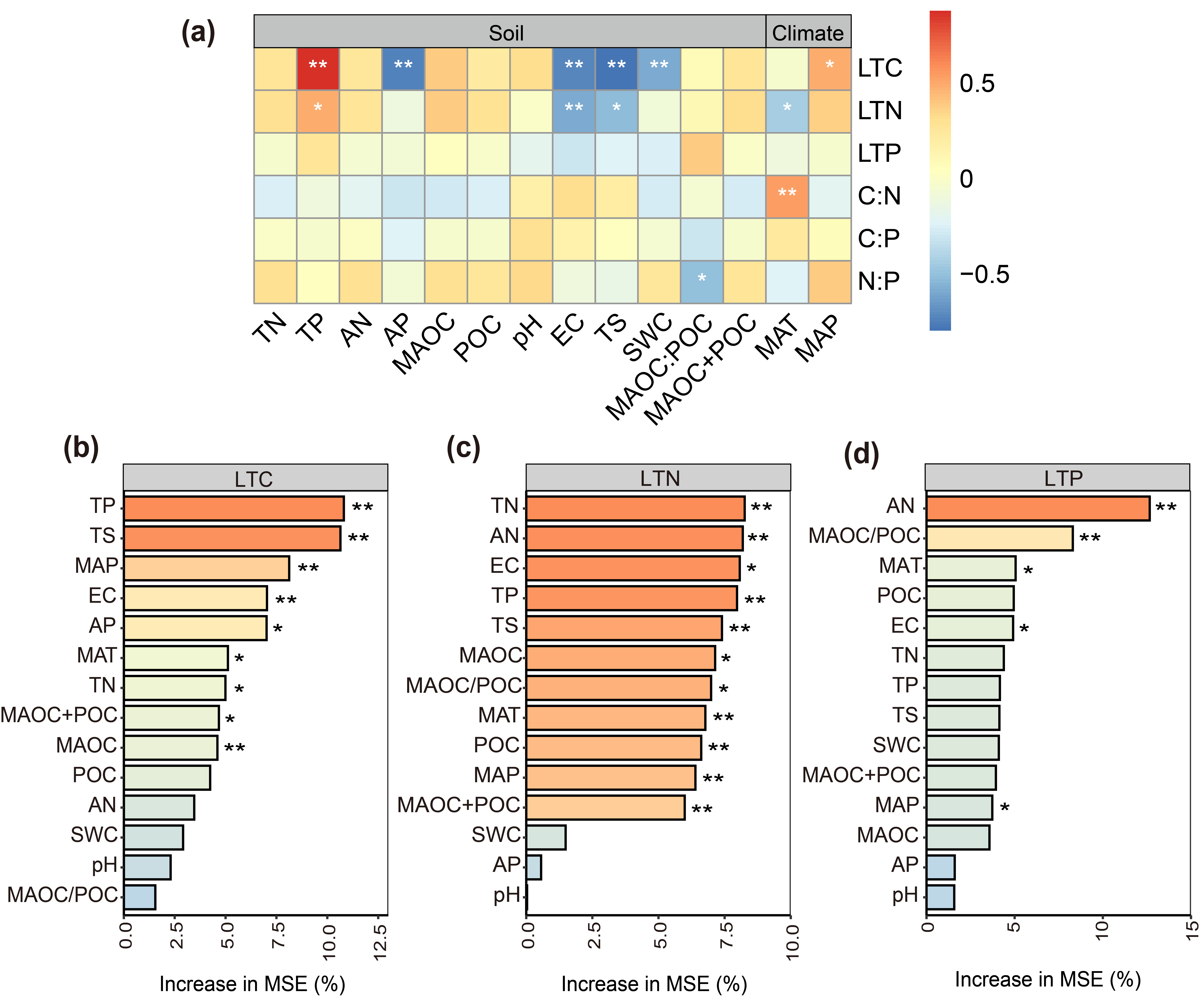


Fig.S2 Correlations between leaf nutrient contents and their stoichiometric ratios in plant communities with soil and climatic factors are shown in figure (a). Figure (b), (c), and (d) display the key environmental variables influencing variations in LTC, LTN and LTP, respectively, as identified and ranked by the random forest model. * and ** represent the significance at 0.05 and 0.01 level.

Table S1 Overview of sampling sites along the elevational gradient in the study area

| Altitude (m) | Longitude | Latitude | Vegetation type | Dominant species | MAT (°C) | MAP (mm) |
| --- | --- | --- | --- | --- | --- | --- |
| 1960 | 80°44′29.64″E | 36°36′27.96″N | Semi-shrub desert | *Reaumuria soongonica, Calligonum roborowskii*, *Zygophyllum rosowii, Halogeton glomeratus* | 10.87 | 91.0 |
| 2448 | 80°33′52.84″E | 36°22′59.33″N | Semi-shrub desert | *Reaumuria soongonica*, *Sympegma regelii*, *Grubovia dasyphylla* | 3.29 | 111.3 |
| 2746 | 80°22′45.69″E | 36°16′03.70″N | Desert steppe | *Seriphidium rhodanthum*, *Reaumuria soongonica* | 6.42 | 133.5 |
| 2905 | 80°19′45.46″E | 36°14′38.40″N | Desert steppe | *Seriphidium rhodanthum*, *Stipa roborowskyi, Allium przewalskianum* | 4.78 | 229.5 |
| 3248 | 80°15′47.10″E | 36°12′15.17″N | Alpine steppe | *Seriphidium rhodanthum*, *Stipa roborowskyi*, *Allium przewalskianum*, *Leontopodium nanum* | 2.53 | 285.6 |
| 3548 | 80°14′14.64″E | 36°09′48.45″N | Alpine steppe | *Festuca rubra*, *Leontopodium nanum*, *Potentilla chinensis*, *Stipa capillata* | 1.26 | 344.3 |
